# Effects of Processing Variables of Extrusion–Pultrusion Method on the Impregnation Quality of Thermoplastic Composite Filaments

**DOI:** 10.3390/polym12122833

**Published:** 2020-11-28

**Authors:** Cahyo Budiyantoro, Heru S.B. Rochardjo, Gesang Nugroho

**Affiliations:** 1 Department of Mechanical and Industrial Engineering, Universitas Gadjah Mada, Yogyakarta 55281, Indonesia; cahyo_budi@umy.ac.id (C.B.); gesangnugroho@ugm.ac.id (G.N.); 2 Department of Mechanical Engineering, Universitas Muhammadiyah Yogyakarta, Yogyakarta 55183, Indonesia

**Keywords:** carbon fibre-reinforced polypropylene filament, extrusion–pultrusion, impregnation die, liquid nitrogen, response surface method, pull-out test, silane-coupling agent, interfacial shear strength

## Abstract

Carbon fibre-reinforced polypropylene composite filaments were fabricated via the extrusion–pultrusion method. One of the important factors influencing composites’ filament processability and structural properties is the impregnation quality, which can be represented by interfacial adhesion between the matrix and fibre. To improve the interfacial shear strength (IFSS) of the filament, four processing variables—melt temperature, pulling speed, number of pins in the impregnation die and fibre treatment—have been optimised using the Box–Behnken response surface methodology (RSM). Analysis of variance (ANOVA) was conducted to evaluate the linearity of the response surface models. Three levels were set for each independent variable. The melt temperature was varied at levels 190, 210 and 230 °C, while the pulling speed was set at three levels, namely, 40, 47 and 50 cm/min. The number of spreader pins was varied at 1, 2 and 3 pins, and there were three variations of the fibre treatment, namely, vinyltrimethoxysilane (VTMS), γ-aminopropyltriethoxy silane (APTS) and liquid nitrogen. Twenty-seven experimental runs were conducted, and a significant regression for the coefficient between the variables was obtained. The filament IFSS was measured by a customised pull-out test, and its surface morphology was characterised using a scanning electron microscope. ANOVA showed that fibre treatment significantly affected the IFSS due to their surface roughness, followed by pulling speed and melt temperature in quadratic order. Liquid nitrogen is recommended for carbon fibre treatment because of the high surface roughness, thereby providing a better matrix–fibre bonding effect. The results demonstrated that a melt temperature of 190 °C, pulling speed of 40 cm/min, three spreader pins and treatment of the fibre with liquid nitrogen afforded the optimum impregnation quality. It is important to keep a reasonable low processing temperature to obtain the geometrical stability of the product.

## 1. Introduction

Nowadays, the commercial applications of carbon fibre-reinforced thermoplastics (CFRTPs) are increasing owing to the advantageous properties of carbon fibres (CFs), such as high strength-to-weight ratio, damping capability and rigidity [1]. One way to prepare raw composite materials is to employ the extrusion–pultrusion method, which combines the working principle of extrusion and pultrusion to produce CFRTP filament. It provides a uniform filament shape with constant fibre content. The quality of CFRTPs is indicated by the final product’s mechanical properties, which are mainly influenced by the fibre–matrix interactions. This factor can be thermally and mechanically driven by optimising processing parameters and die design. However, both the CF and thermoplastic contribute to weak interfacial bonding. CF has low adsorption and wetting when interfaced with most types of thermoplastics. The surface of CF is nonpolar; therefore, the interfacial shear strength (IFSS) between the fibre and matrix is poor, and consequently the mechanical performance of the composite is not optimal [2].

Moreover, impregnation exists between thermoplastic matrices and fibre bundles in a composite system because of the high thermoplastic melt viscosity (500–5000 Pa·s) [3]. Low impregnation quality may deteriorate the mechanical properties of the composite [4,5]. To increase the surface energy and roughness, surface treatment is suggested to increase the surface area and contact points, micropores or surface grooves on the CF surface. In general, three methods can be used for fibre surface treatment: plasma, chemical and electrochemical treatments [5]. Coupling agent (CA) treatment is the most popular chemical method because it provides ease of use on a laboratory scale and increases bonding properties without degrading fibre properties [5,6]. Silane CA forms the alkoxysilane group that reacts with the hydroxyl group after hydrolysis. CFs can be modified by surface oxidation as well. Yuan et al. [7] oxidised the CF surface by heating CF in 40 wt.% H_2_SO_4_ and 15wt.% KClO_3_ at 85 °C for 30, 60, 90 and 120 min. Thereafter, the oxidised fibres were thoroughly rinsed with deionised water and dried at 50 °C. The results indicated that CF surface treatment by oxidation and (γ-aminopropyl) triethoxysilane (APTS) improved the surface chemical activity and surface roughness and increased the surface area of the fibre. Wen et al. [8] applied a two-step fibre treatment—electrochemical oxidation followed by a silane CA. After oxidation, CFs were immersed in a silane solution made by mixing 5% KH550 CA with a mixture of 5% distilled water and 90% ethanol; before immersing CFs, the solution was mechanically stirred for 1 h. Furthermore, the fibre drying process was performed in an oven at 100 °C. The application of KH550 CA to the fibre surface yields a remarkable improvement in fibre surface energy and the wetting effect between the CFs and polymer matrices. The tensile strength of the treated fibres also significantly increased. In addition to CAs, some researchers used liquid nitrogen for fibre treatments. The effects of liquid nitrogen treatment on the interfacial bonding of carbon fibre-reinforced polypropylene (CFRP) and mechanical properties of CF were investigated by Kim et al. [9]. The composite had a tensile strength value of 70 MPa, Interlaminar Shear Strength (ILSS) of 9.5 MPa and impact strength of 7.8 kJ/m^2^.

As mentioned above, the impregnation quality is one of the main issues in CFRTP production [10]. By covering all individual filaments of the fibre with a matrix, high-performance composite characteristics can be achieved. In the extrusion–pultrusion system, the wetting of fibre can be improved by pin-assisted die design and fibre movement mechanism in the plastic melt that accommodates sufficient melt impregnation. In a pin-assisted melt impregnation die, the impregnation quality is closely related to the following parameters; melting temperature of the plastic, pressure, pin number, pin dimension, pin layout, fibre tension and fibre pulling speed [11]. During impregnation, fibre bundles must be stretched out sideways so that each strand moves aligned, does not overlap one another and can be evenly wetted by the matrix. Fibre stretching can be mechanically performed by passing the fibre through several spreader pins attached to the die; the pins that are not aligned make the fibre bundle widely spread as it passes through the pins. The pin cross-curve radius and the height difference between the pins affect the stretching of fibres that pass through it [12]. In the case of a viscous plastic material, such as polypropylene (PP), the penetration of the resin into the fibre is more difficult. According to Gayman et al. [13], the impregnation rate was reduced by increasing the pin diameter. Fibre tension on the pins changes with pulling speed, and by increasing the fibre tension, the permeability of the fibre can be decreased. Kabeel et al. [14] studied the melt impregnation of continuous CFR-PA 66. They developed an impregnation system with a series of parallel pins; the fibre passed along a set of parallel pins, and the impregnation took place in the contact area. The exit die could be used to control the fibre content. Marissen et al. [15] developed melt impregnation technology by passing the glass fibre bundle on five conical spreading pins in the polypropylene matrix. Nygard and Gustafson [16] compared the efficiency of different melt impregnation methods—pin-assisted methods, a crosshead impregnation die, use of a slit die and different vibration methods. The radial slot impregnation method afforded the best overall impregnation efficiency; a high degree of impregnation could be held at the haul off maximum speed of 10 m/min. The quality of impregnation depends on the contact time between the fibre bundle and the impregnation bar. A higher speed generates a higher pulling force that can increase values above the maximum fibre bundle strength. In addition, it decreases the contact time, consequently reducing the quality of impregnation. These methods can improve impregnation quality; however, this technology is complex for application in mass production and retains the plastic melt in the container. The proposed method considers only impregnation without observing other quality indicators, such as fibre–matrix interactions.

Moreover, the filament composite manufacturing process involves a combination of many parameters and is not influenced by the factors mentioned earlier. The selection process of a proper combination of parameters is crucial as it highly influences the product quality. Nonetheless, to obtain the optimum process parameters for composite products, one must often rely on time-consuming trial-and-error methods. The optimal process parameters can be determined by response surface methodology (RSM), which is efficient and convenient for experimental design. RSM involves statistical and mathematical methods that are useful for analysing and modelling problems wherein a target response affected by several variables is to be optimised. These designs can fit a second-order prediction equation for the response. Parametric optimisation research using the Taguchi design of experiment was conducted by Xian et al. [11]; the optimised parameters were melt temperature, roving pretension, pulling speed and the number of impregnation pins, while the target response was the degree of impregnation. The results showed that the pulling speed has the most powerful influence on the degree of impregnation, followed by the melt temperature and the number of pins. Ren et al. [17] analysed the effect of pulling speed and the number of pins on the fibre fracture in thermoplastic-based composite melt impregnation. They applied RSM to obtain the optimal parameters and compared it with a mathematical model. They found that the number of pins yielded the most significant factor in the fibre fracture. High-quality impregnation can be achieved using low pulling speed as a relatively high melt temperature negatively impacts impregnation quality. Chen et al. [18] used the RSM, Taguchi method and hybrid genetic algorithms–particle swarm optimization (GA-PSO) to identify the combination of the optimal process variables of the injection moulding machine (melt temperature, packing pressure, injection velocity, cooling time and packing time) to obtain minimum shrinkage and warpage. This study enhances the stability of the injection moulding process by reducing injection costs and the time required for the trial. Fu et al. [19] performed RSM and analysis of variance (ANOVA) to analyse the effects of melt temperature, screw speed and the recycled component on the melt pressure, mass output, screw torque and temperature increase at the die in a PP blend system. The study presented quantitative data for single-screw extrusion and showed the importance of a design of experiment (DoE) method to predict the variety of potential processing conditions for production operations.

In this study, CFRP composite filaments were manufactured via the extrusion–pultrusion method. This study aims to obtain a composite filament with consistent high impregnation quality. The impregnation quality was quantified using stress-based approaches by measuring IFSS. Processing variables affecting the process, such as melt temperature, pulling speed, number of spreader pins and fibre treatment, were optimised using Box–Behnken RSM. IFSS tests and microscopic analyses were performed to determine the impregnation quality of the filaments.

## 2. Materials and Methods

### 2.1. Materials

In this study, a Cosmoplene AW564 high-impact polypropylene copolymer was used as the matrix material. It is produced by The Polyolefin Company (Singapore) Pte Ltd., Singapore. It is medium-flow, high-stiffness and high-impact copolymer grade material [20]. As a reinforcing fibre, CF T700SC 12K [21], made by Toray Composite Materials Americe, Inc., Tacoma, WA, USA, was used. Three types of materials were used in fibre treatment: vinyltrimethoxysilane (VTMS), γ-aminopropyltriethoxy silane (APTS) and liquid nitrogen. Both VTMS and APTS are silane CAs with silicon and hydroxyl groups supplied by Hangzhou Jessica Chemicals Co., Ltd, Hangzhou, China. Silane CAs are widely used in composite materials to enhance the compatibility between the polymer and inorganic substance, considerably improving the mechanical properties of composite products [22]. Table 1 lists the properties of the incorporated materials.

### 2.2. Pulling Speed and Melt Temperature of the Extrusion–Pultrusion Method

**Figure 1:**
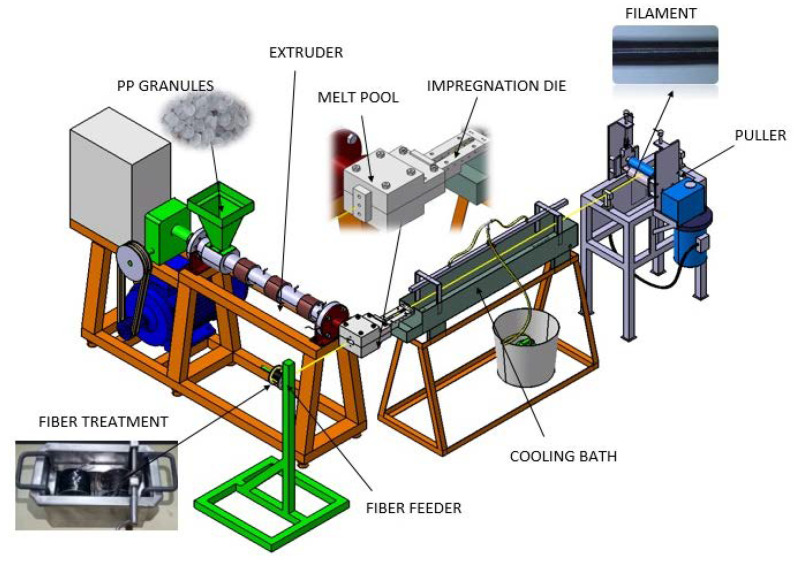
Schematic of the extrusion–pultrusion machine.

The extrusion–pultrusion method was developed to produce thermoplastic composite filaments with a cylindrical cross-sectional diameter of 2–3 mm. In principle, this method comprises two units: an extrusion unit and a pultrusion unit. The extrusion unit works to melt plastic pellets through heating and shearing in the barrel. The plastic melt is pushed out of the barrel, temporarily stored in the melt pool and then moved towards the impregnation die. From the perpendicular direction to the die, the treated CF bundles are continuously pulled by the pultrusion unit, spread by several pins and impregnated by plastic melt inside the die. The hot filament is then cooled down by water spraying in the cooling bath and completely solidified. Figure 1 shows the schematic of the extrusion–pultrusion system.

Two main parameters of this method that influence the production results are the melt temperature of the plastic that is adjusted in the extrusion unit and the pulling speed adjusted in the pultrusion unit. As per the material supplier’s recommendations, the process temperature range for polypropylene is 190–230 °C. Fu et al. [19] used the barrel temperature in the 180–240 °C range for polypropylene extrusion; thus, the selected temperature in this study is reasonable. Based on initial experiments, the ideal pulling speed ranges from 40 to 54 cm/min. The extruder screw diameter is 38 mm, and the length is 760 mm so that at a constant screw rotation of 10 rpm, the volume throughput generated by extrusion is 4.2 Kg/h.

### 2.3. Impregnation Die Design

**Figure 2:**
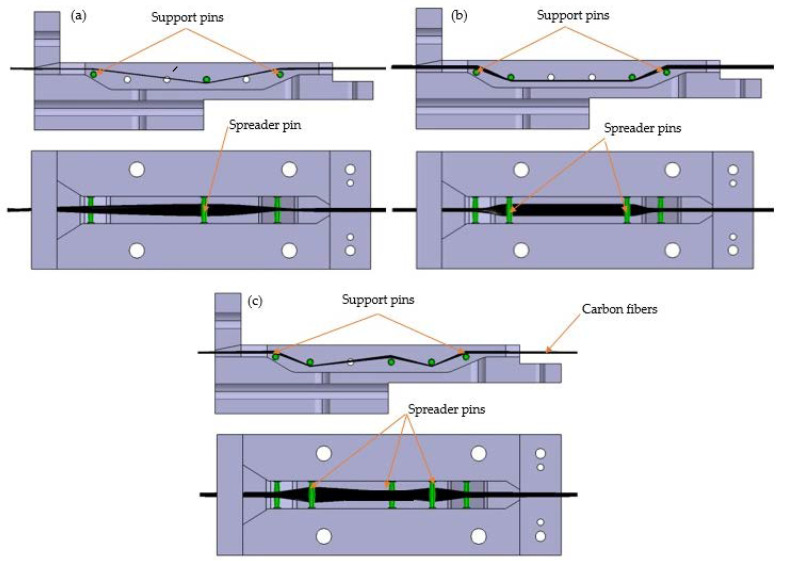
Various impregnation dies designs used in this study: (**a**) Pin layout 1—one spreader pin, (**b**) Pin layout 2—two spreader pins, and (**c**) Pin layout 3—three spreader pins.

The design of the impregnation die was also considered as it influences the filament quality. The die design should improve the spreading condition of the fibre bundle to improve impregnation quality. In this case, the die design relies on the number of pins and pin configuration. Figure 2 shows the three variations of die design used in this study; the variations are distinguished according to the number of fibre spreader pins located between the two support pins (one pin, two pins and three pins). The die had six holes where the pins could be inserted in many arrangements based on spacing and position. The melt impregnation chamber’s length was 160 mm, and the volume of the melt pool was 34.3 cm^3^. The fibre bundle was pulled out through the die and passed through the pins.

**Figure 3:**
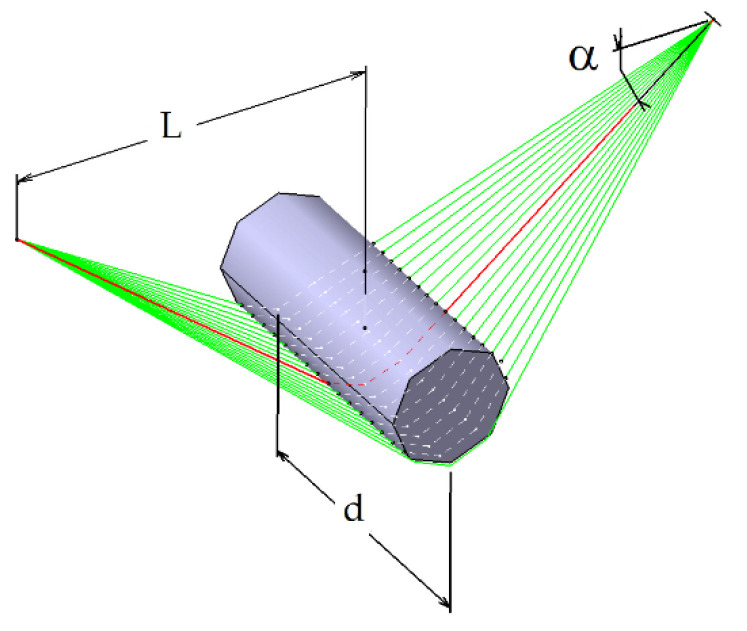
Fibre bundle spreading model.

The bundle of fibre was spread depending on the curvature radius of the pins and the distance of the pins. The fibre was compressed by the pin surface and spread sideways; then, the contact area between the fibre bundle and matrix increased. A parameter model of the fibre bundle spread width, fibre bundle cross section, the distance between adjacent pins and other related parameters was proposed by Wilson [23], as shown in Figure 3. Impregnation dies with a spreader pin position and geometry refer to Wilson’s theoretical calculations Equation (1):

(1) $$d=\sqrt[{3}]{\left(12AL\cos\alpha \right)}$$

where d is the fibre bundle spreading width and *A* is the cross section of the fibre bundle, which is connected to the fibre bundle cross section shape. Furthermore, *L* is the distance between the fibre bundle and the adjacent pin’s tangent point, and *α* is the contact angle between the fibre bundle and pin.

### 2.4. Fibre Surface Treatment

**Figure 4:**
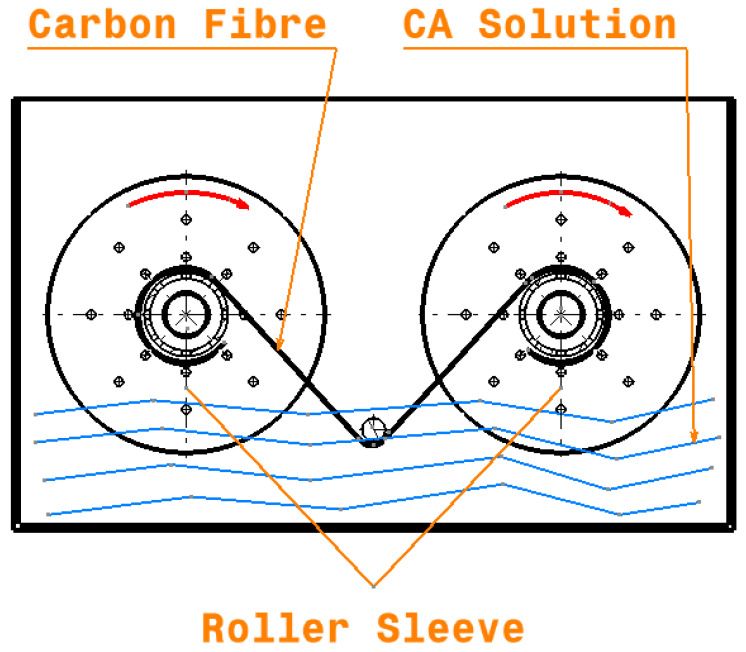
Fibre immersion bath.

CFs were prepared via three different surface treatments before the manufacturing process. The first treatment was fibre immersion in a CA solution composed of 1 wt.% VTMS and 99 wt.% distilled water; this is referred to as Treatment 1. The second treatment, Treatment 2, involved immersing the fibres in a mixing solution of 1 wt.% APTS and 99 wt.% distilled water. The pH of each solution was reduced to 4.2 with the help of 0.5% acetic acid. The solution was mechanically stirred for 30 min to ensure a complete silane hydrolysis process [6,24]. CF coils were immersed in the solution to obtain an even penetration of the solution over the entire surface of the fibres; the fibres were wound slowly from one roller to another through a pin submerged in the immersion bath, as shown in Figure 4. The fibre winding process in this solution was performed continuously for 20 min. Furthermore, the fibres were also washed in running water by the winding process. Fibre coils were dried in hot air at 80 °C for 1 h to ensure that the fibres do not agglomerate.

The third fibre treatment method, Treatment 3, was a cryogenic treatment by nitrogen immersion. The fibre coils were immersed and gently rotated in liquid nitrogen (−196 °C) for 10 min [9]. Liquid nitrogen [25,26] surface treatments are recommended methods to enhance the fibre surface roughness, and therefore improve mechanical interlocking and adsorption interaction between the polymer matrix and CF.

### 2.5. Design of Experiment

In this study, the Box–Behnken RSM was used to investigate the effect of process variables on the impregnation characteristics indicated by the fibre–matrix interfacial strength. The experimental design data analysis was performed with the help of Design-Expert version 12 software (Stat-Ease, Inc., Minnesota, United States). The 3-level 4-factor design was used in this analysis, and it required 27 experiments to run. Four defined independent variables were A, melt temperature (190–230 °C); B, pulling speed (40–54 cm/min); C, number of pins (1–3); and D, fibre treatment (3 types of treatment). In this case, A, B and C are numerical variables, while D is a comparable categorical variable. Furthermore, Table 2 presents the coding for each factor level used in Software Design Expert 12 along with the actual level. The relation between the actual level and the coding is as follows [27],

(2) $$x_{i}=\frac{\left(X_{i}-X_{\min}\right)}{\Delta X_{i}},\;i=1,\;2,\;3$$

where $x_{i}$ the ith level’s encoding value, $X_{i}\;$is the actual level value, $X_{\min}\;$is the lowest level value and $\Delta X_{i}\;$is the difference for each level.

**Figure 5:**
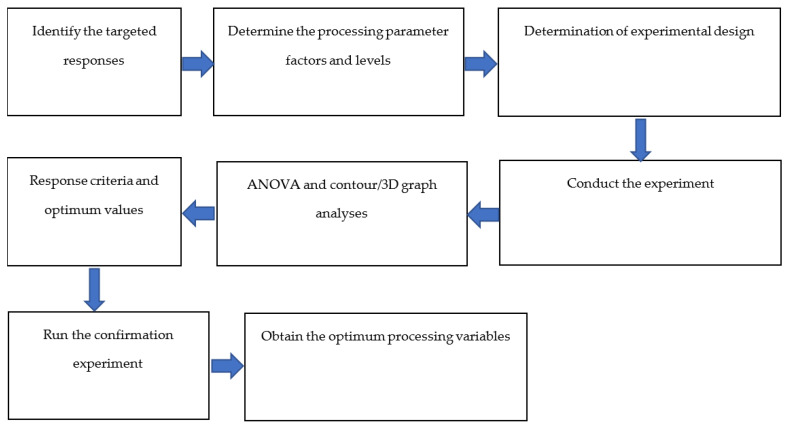
Experimental sequence.

The complete design of the experiment determined by the Box–Behnken RSM is presented in Table 3, with 27 experimental runs comprising 24 distinct runs and three replications. The experimental sequence is described in Figure 5. Finally, ANOVA, regression analysis and contour plotting were used to evaluate the optimum conditions for the IFSS as an indicator of impregnation quality.

### 2.6. Testing Methods

**Figure 6:**
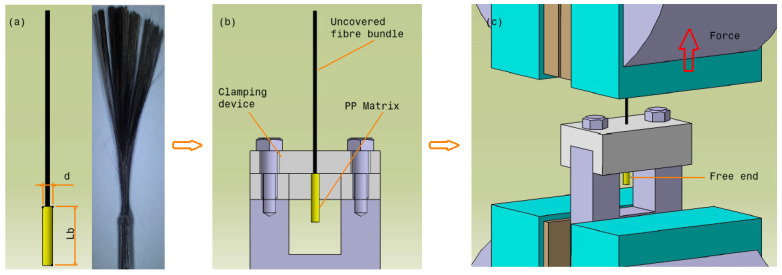
Pull-out testing system: (**a**) test specimen, (**b**) clamping position and (**c**) test position.

No standard test method exists for measuring the IFSS between the fibre bundle and thermoplastic matrix. The single-fibre pull-out (SFPO) method is the most common test method to measure IFSS, but the bundle pull-out test is more physical and closer to the real application. However, SFPO test results are not relevant to industrial applications [28]. This is because, first, during the fracture process, fibres are pulled as a bundle and not individually [29], and second, to establish the mathematical models, a specially designed model with ideal geometrical conditions are needed, but this model cannot be used in manufacturing. In this study, the IFSS was measured by a pull-out test adopted from Zhandarov et al. [30], Chandran et al. [31], Cech et al. [32] and Sakai et al. [33]. The test involved pulling a partially embedded fibre bundle out of matrix envelopes. All specimens were conditioned at 23 °C and 50% relative humidity (R.H.) for 24 h prior to testing. The test was performed on a Zwick/Roell universal testing machine at a room temperature of 23 °C and 50% R.H. with a crosshead moving speed of 2 mm/min; maintaining a low strain rate avoids compliance-related problems. Three samples were tested for each parameter combination; then, the average IFSS values were determined. Before the IFSS test, the filament was cut to a length of 50 mm. The thermoplastic resin covering the fibres was removed, leaving only a bonding length (*l*_b_) in the 3–5 mm range (Figure 6a). The bonding length range followed the manual cutting results, and the length was used in the IFSS calculation. The bond length of the matrix over the fibre bundle is a significant parameter. The bond length should not be too long to ensure that the fibre does not break before the complete failure of the interface occurs. The critical bond length (*l*_b_) can be determined using the Equation (3) [31]:

(3) $$\;l_{b}=\frac{\sigma_{f}\times d}{4\tau }$$

where *σ*_f_ indicates the ultimate fibre tensile strength at break, *d* is the fibre bundle diameter, and the shear strength of the bond is represented by *τ*.

A special clamping device was used to hold the specimen’s matrix, as shown in Figure 6b. In this testing system, the composite sample was placed upright in a clamping device, and the uncovered fibre bundle was gripped by a V-jaw grip (Figure 6c). The uncovered fibre bundle of the specimen was considered the loaded end. The IFSS (*τ*) can be determined as the maximum applied load divided by the contact area using the Equation (4) [34]:

(4) $$\tau =\frac{F}{\pi d\times L_{b}}$$

where *F* is the measured maximum debonding force, d is the average fibre bundle diameter and *L*_b_ is the bond length. Here, the bundle diameter can be measured only as an average value from the product sampling. The clamping device is optimally designed to minimise any frictional force with the fibre or resin.

A scanning electron microscope (SEM) on the pulled-out area of the specimen was used after the pull-out test to analyse and compare the failure condition; the pulled-out area of the specimens was sputtered with gold. The observed area of the specimen was embedded in an epoxy resin and polished using fine sandpaper.

## 3. Results and Discussion

### 3.1. IFSS

**Figure 7:**
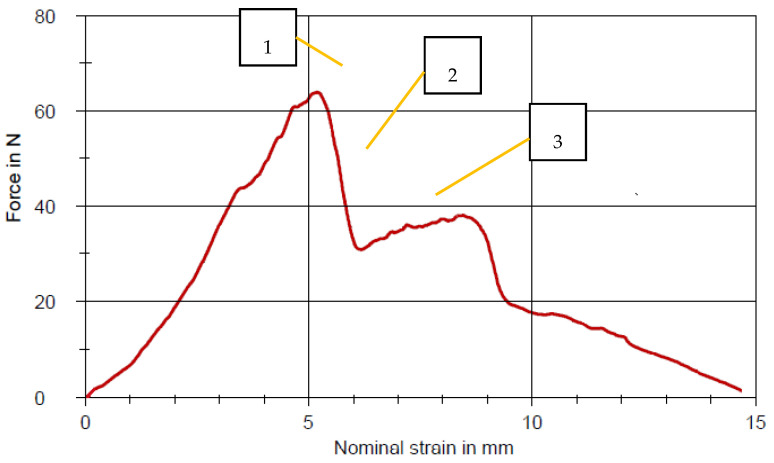
Typical force versus fibre displacement curve for the pull-out test.

IFSS was calculated using the maximum force recorded in the test corresponding to the start of unstable crack propagation. Its value is substantially influenced by friction in debonded regions. This maximum load is divided by fibre displacement when pulled out of the matrix. The typical force–displacement curve taken from the pull-out test of the specimen performed without fibre treatment and spreader pin is shown in Figure 7. The figure shows the stages where the fibre bundle experiences a displacement towards the matrix holder due to the applied tensile force: (1) pull-out leads to debonding, (2) propagation of debonding front and (3) frictional sliding occurs once the debonding and propagation are complete.

The test results of the IFSS of CFRP filament composites with different processing variables are summarized in Table 4. The IFSS results were taken from the average IFSS value of three specimens in each trial. The results of Trial 26 show that the highest IFSS can be achieved by treating fibre with liquid nitrogen, applying a melt temperature of 210 °C, pulling speed of 40 cm/min and using two spreader pins. The lowest bonding strength occurs at a melt temperature of 210 °C, pulling speed of 54 cm/min and using two spreader pins as well as VTMS treatment. The IFSS calculation was done using Equation (3); here, the average diameter of the fibre bundle was 0.85 mm, and the bond length of each specimen was in the 3–4 mm range as a result of manual cutting.

The 27 experiments that have been conducted, as shown in Table 3, were based on an orthogonal array randomly generated by the software; therefore, all the parameter combinations could not be represented. Further analysis and optimization are necessary to find the possibility of higher IFSS values.

### 3.2. ANOVA Analysis and Model Fitting for IFSS of CFRP Composite Filament

Table 5 displays the ANOVA for the IFSS quadratic response surface model; this method is used to identify the significance of the model and its parameters based on the F test, which identifies the significance of the obtained results. As shown in Table 5, the *p* value of the regression model is <0.05, which means that the model is significant at a significance level α = 5%. There is only a 3.27% probability that such a high F value will occur due to noise. The factor coefficients that have a significant effect on the model are B (pulling speed), D (fibre treatment) and A^2^ because the p value is <5%. A “Lack of Fit F value” of 7.94 shows that the Lack of Fit is not significant compared to the pure error. An 11.70% chance exists that a “Lack of Fit F value” this large could occur due to noise, and an insignificant lack of fit is good. Significant lack of fit indicates that there might be contributions in the regresses–response relationship that are not accounted for by the model. The insignificant *p* value thus indicates that the model was good and fitted well to the experimental data.

Furthermore, the model’s validation can be performed based on the coefficient of determination (R^2^) and the fitting of the F model. Table 6 shows the statistical measurement of the goodness of the model. The R^2^ value is 77.66%, indicating that the model is good enough with an adjusted R^2^ value of 51.59%. A negative “Pred R-Squared” implies that the overall mean may be a better predictor of the response than the current model. “Adeq Precision” determines the signal-to-noise ratio. A ratio of more than 4 is optimal. Here, a ratio of 6.292 indicates an effective signal. The model can be used to navigate the design space.

### 3.3. Diagnostic Plots of CFRP Filament Composite

**Figure 8:**
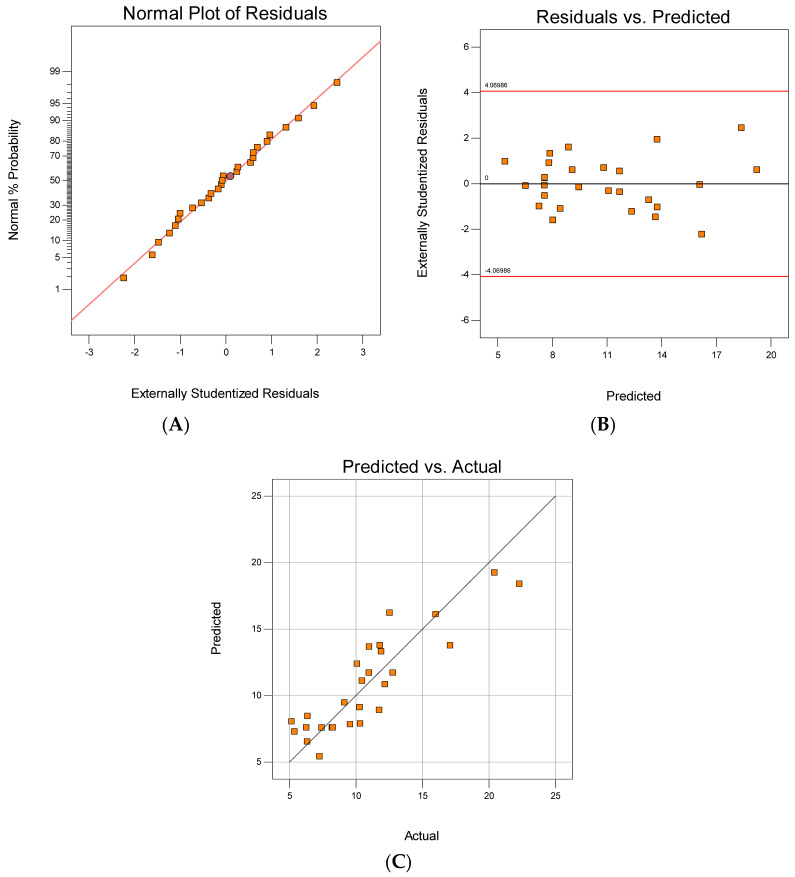
Diagnostic plots: (**A**) normal plot of residuals, (**B**) residuals vs. predicted and (**C**) predicted vs. actual.

The adequacy of the model can be calculated by applying diagnostic plots, such as the predicted versus actual values, normal probability and residuals versus fitted values plots. The diagnostic plots of this study are shown in Figure 8. Figure 8A shows a typical normal versus residual probability distribution. The residual plots fall near the linear line in the figure, indicating that there is a good fit with the normal distribution [35]. On the other hand, Figure 8B presents a plot of residuals versus predicted values, showing that the data points are randomly scattered throughout the plot without exceeding the upper and lower boundary lines confirming the proposed model’s accuracy. In general, the studentised residuals should lie between +3 and −3 [36]. Figure 8C presents the predicted and actual response values, which appear to agree because the data points are scattered around a linear line. The model’s residual plots are randomly distributed without any patterns. This result also indicates a good prediction of the adequacy of the quadratic models.

**Figure 9:**
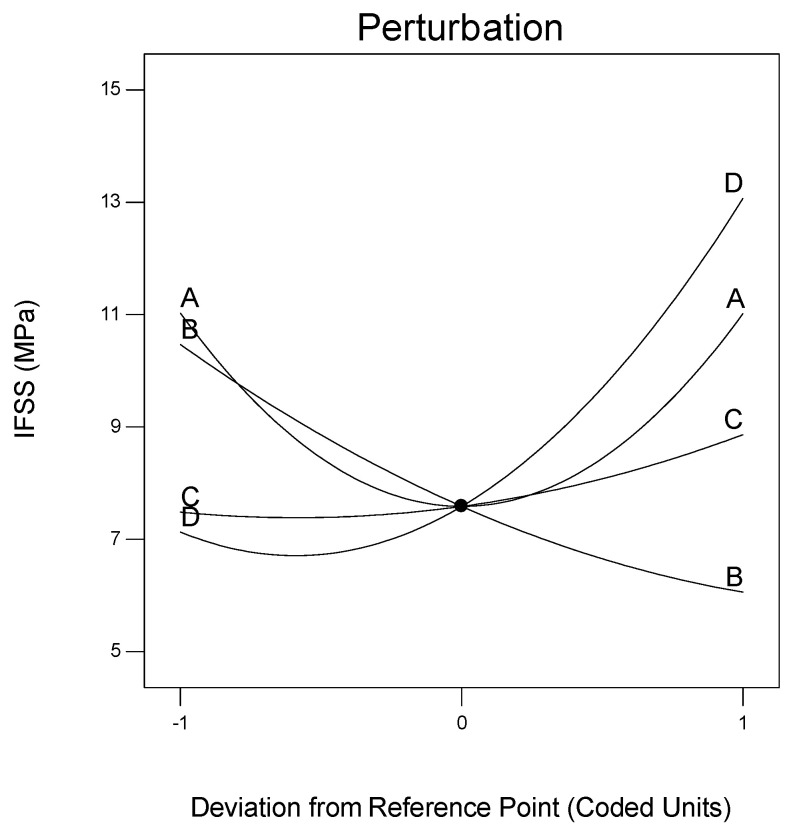
Perturbation plot showing the influence of all factors on IFSS at the midpoint of the design space.

The perturbation plot in Figure 9 shows the effect of processing conditions on IFSS. The plot also depicts the change in response as each variable moves from the centre of the design space (A = 210 °C, B = 47 cm/min, C = 2 and D = 2). Here, the pulling speed harms IFSS because the IFSS value decreases as the pulling speed increases. In contrast, the number of spreader pins and fibre treatment factors positively affects the IFSS value. The results of the regression coefficient test show A have a quadratic and not a linear effect on IFSS.

### 3.4. Response Surface Plots

**Figure 10:**
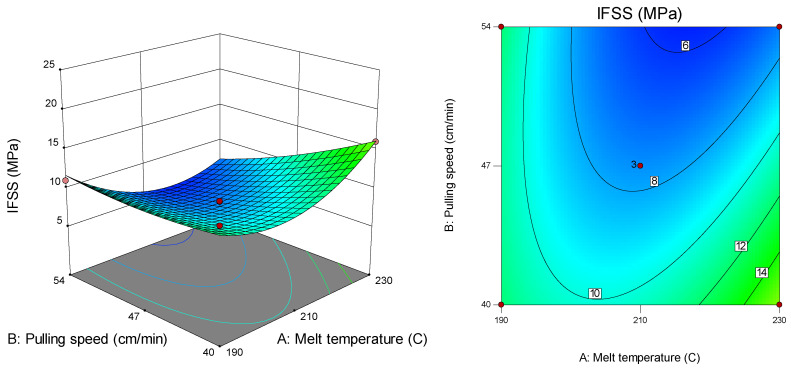
Surface and contour plots of IFSS versus melt temperature and pulling speed.

Figure 10 presents a surface plot and a contour plot that is suitable for the interaction effect between melt temperature (A) and pulling speed (B) on IFSS for two pins (C) and VTMS-treated fibre (D). From the plot, the highest IFSS value (16.0 MPa) was recorded at a melt temperature of 230 °C and pulling speed of 40 cm/min. Conversely, the lowest IFSS (5.4 MPa) was recorded at a melt temperature of 230 °C and pulling speed of 54 cm/min.

**Figure 11:**
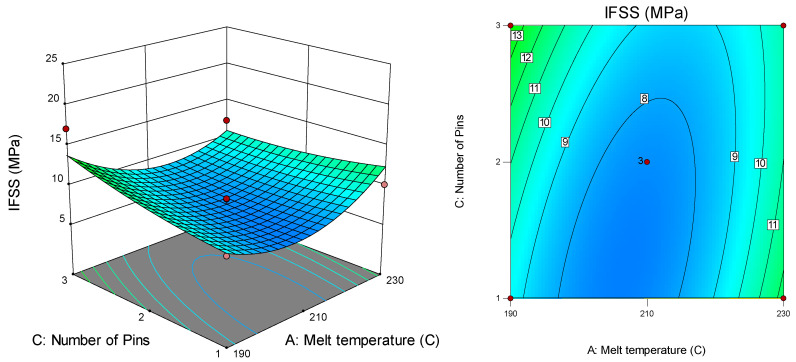
Interaction effect between melt temperature and number of pins on IFSS.

**Figure 12:**
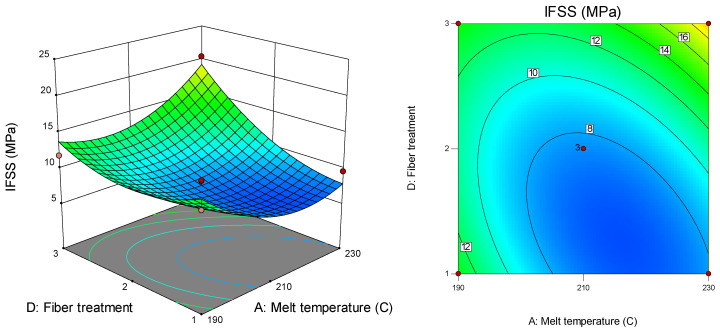
Interaction effect between melt temperature and fibre treatment on IFSS.

Figure 11 describes the surface and contour plots that are suitable for the interaction effect between melt temperature (A) and the number of pins (C) on IFSS at a pulling speed of 47 cm/min for fibre treatment with VTMS. From the plot, the highest IFSS value (17.1 MPa) was recorded at a melt temperature of 190 °C for three pins. In contrast, the lowest IFSS value was 6.3 MPa, recorded at a melt temperature of 210 °C for two pins. Figure 12 presents the surface and contour plots for the interaction effect between melt temperature (A) and fibre treatment (D) on IFSS at a pulling speed of 47 cm/min for two pins.

The highest IFSS value (20.4 MPa) was recorded at a melt temperature of 230 °C for the liquid nitrogen-treated fibre, while the lowest IFSS (6.3 MPa) was recorded at a melt temperature of 210 °C for the APTS-treated fibre.

**Figure 13:**
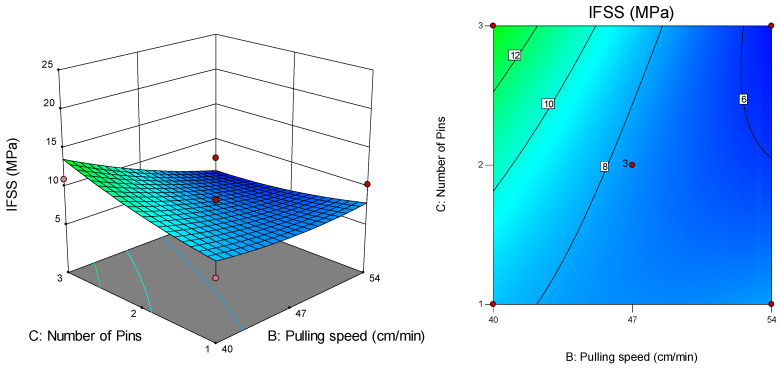
Interaction effect between pulling speed and number of pins on IFSS.

Figure 13 shows the surface and contour plots suitable for the interaction effect between pulling speed (B) and number of pins (C) on IFSS at a melt temperature of 210 °C for the APTS-treated fibre. The highest IFSS (11.0 MPa) was recorded at a pulling speed of 40 cm/min for three pins, while the lowest IFSS (6.3 MPa) was recorded at a pulling speed of 40 cm/min for two pins.

**Figure 14:**
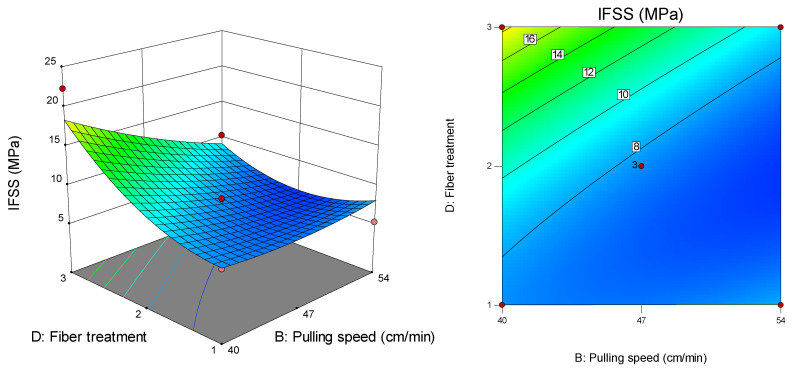
Interaction effect between fibre treatment and pulling speed on IFSS.

Figure 14 presents the surface and contour plots for the interaction effect between pulling speed (B) and fibre treatment (D) on IFSS at a melt temperature of 210 °C for two pins. The highest IFSS (22.3 MPa) was recorded at a pulling speed of 40 cm/min for the APTS-treated fibre, while the lowest IFSS (6.3 MPa) was recorded at a pulling speed of 47 cm/min for the APTS-treated fibre.

**Figure 15:**
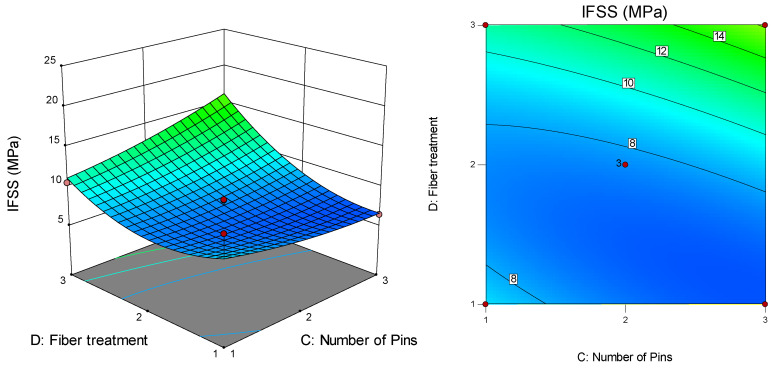
Interaction effect between fibre treatment and number of pins on IFSS.

Figure 15 presents the surface and contour plots for the interaction effect between the number of pins (C) and fibre treatment (D) on IFSS with a melt temperature of 210 °C and pulling speed of 47 cm/min. The highest IFSS value (16.2 MPa) was recorded for three pins and the liquid nitrogen-treated fibre, while the lowest IFSS (6.5 MPa) was recorded for two pins and the VTMS-treated fibre.

### 3.5. Optimum Condition and Confirmation Test

**Figure 16:**
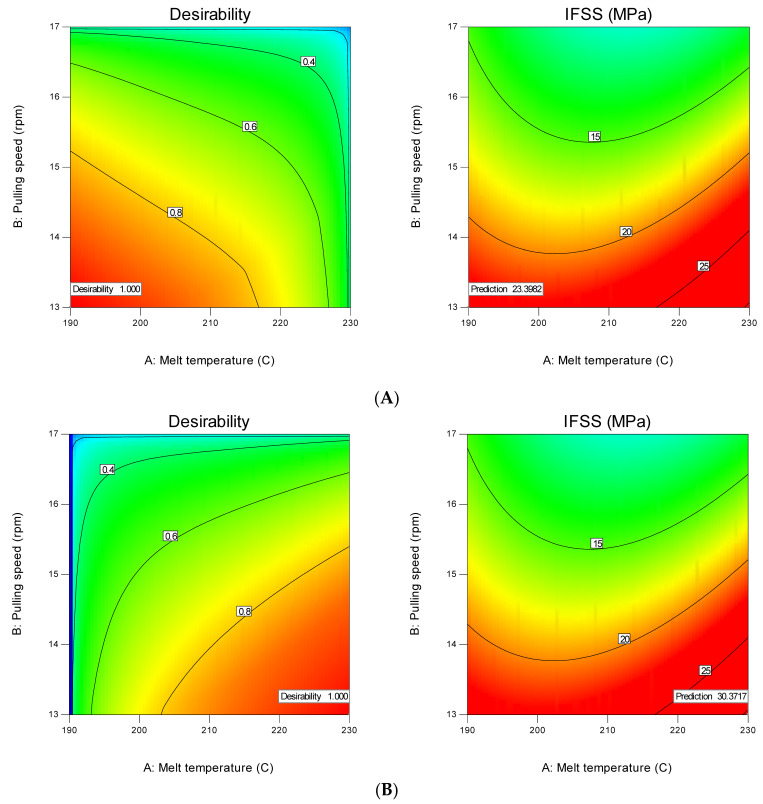
Optimisation solution at (**A**) the minimum melt temperature and (**B**) the maximum melt temperature.

**Figure 17:**
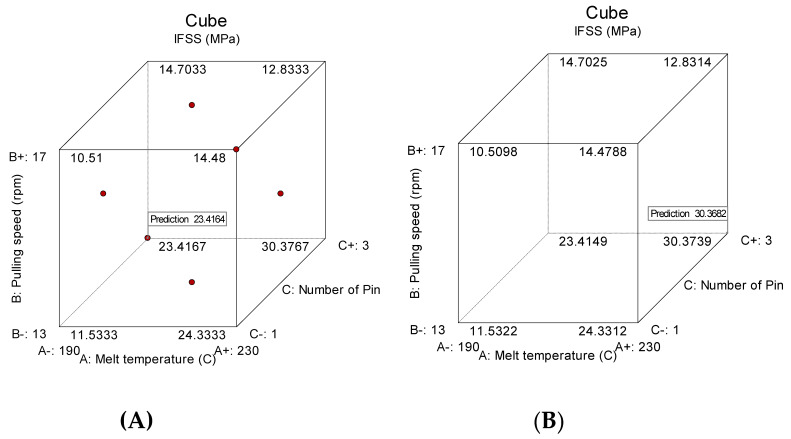
Cube of IFSS prediction at (**A**) the minimum temperature and (**B**) the maximum temperature.

Based on ANOVA and perturbation graphs, fibre treatment (D) is the most influential parameter on IFSS, followed by pulling speed (B) and the second-order term of the melt temperature (A). High IFSS can be achieved by treating the fibre with liquid nitrogen, applying minimum pulling speed (40 cm/min) and using three spreader pins. Meanwhile, the melt temperature should be set to a minimum or maximum to increase IFSS. Optimisation steps need to be taken to describe these assumptions; they help to look for a combination of factor levels that simultaneously meet the response and factor requirements. Figure 16 shows the optimisation analysis at the minimum and maximum melt temperatures. The optimisation analysis at the minimum melt temperature (190 °C) resulted in an IFSS prediction of 23.4 MPa with a desirability level of 1 (Figure 16A). Setting the melt temperature at the maximum position (230 °C) affords a high IFSS result of 30.4 MPa with a desirability level of 1 (Figure 16B). The desirability level is the approach used for factor optimisation in complex systems. The desirability level values lie between 0 and 1. When factors give an undesirable response, 0 is assigned, while 1 refers to the optimal performance for the factors studied [37]. The typical IFSS cube for optimisation is shown in Figure 17; all parameter combination and their IFFS results can be found here.

Three additional experiments were performed using the variables that produced the IFSS prediction to confirm the validity of the predicted optimal response. Table 7 shows the results of the confirmation test; it is more than 90% of the predicted optimal response, indicating that Box–Behnken RSM is an effective method for optimising the extrusion–pultrusion process of CFRP.

**Figure 18:**
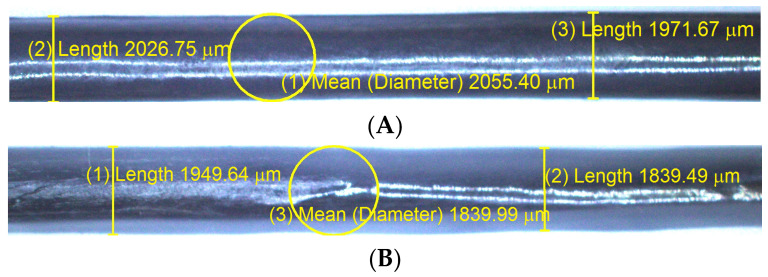
The diameter of the confirmed test specimen: (**A**) melt temperature: 190 °C; (**B**) melt temperature: 230 °C.

A relatively high melt temperature lowers the melt viscosity; then, the plastic melt penetrates between the fibre bundles more easily. With an increased number of spreader pins and low pulling speed, the low viscous plastic melt has sufficient time and space to provide better impregnation quality to fibre bundles that are already in optimal stretching conditions. Low pulling speed also extends the contact time between the spread fibre bundle and pin diameter; then, the plastic melt present in the bundle is redistributed. As previously mentioned, Xian et al. [11], Kabeel et al. [14] and Ren et al. [17] have shown the effects of the pulling speed and melt temperature on the impregnation quality. They found that lower pulling speeds can enhance the quality of impregnation; the melt has enough time to penetrate the fibres. However, in the real production line, low pulling speed implies low production speed. According to Wu et al. [38], the high temperature increased the fibre surface roughness and improved the mechanical interlocking between the matrix and fibre, enhancing interfacial bonding. However, a melt temperature that exceeds the allowable limit can cause the degradation of the thermoplastic material. Ren et al. [12] studied the effect of pin number on the degree of impregnation and the fibre fracture rate; the number of pins used were 3, 4 and 5. They proved that the impregnation degree could be improved by increasing the number of pins, but consequently, the fibre fracture rate also increases. However, in this study, low melt viscosity and three spreader pins reduced the frictional force between the fibre tow and the impregnation pins and minimised fibre fracture. In the above test, the use of high temperatures can provide a better IFSS value. However, some potential problems are present with the application of a high melt temperature. Yang [39] reported that although internal bonding strength increased at high melt temperatures, the risk of thermal degradation of the matrix should be considered. Moreover, the low viscosity matrix can hinder geometric stability. The cross-sectional diameter of the resulting specimen has a large deviation from the die diameter (2 mm) compared to specimens processed at low temperatures, as shown in Figure 18. From the production process, geometric stability is an important consideration to determine product quality. Thus, the optimal process conditions were a melt temperature of 190 °C, pulling speed of 40 cm/min, three spreaders pins and liquid nitrogen treatment.

**Figure 19:**
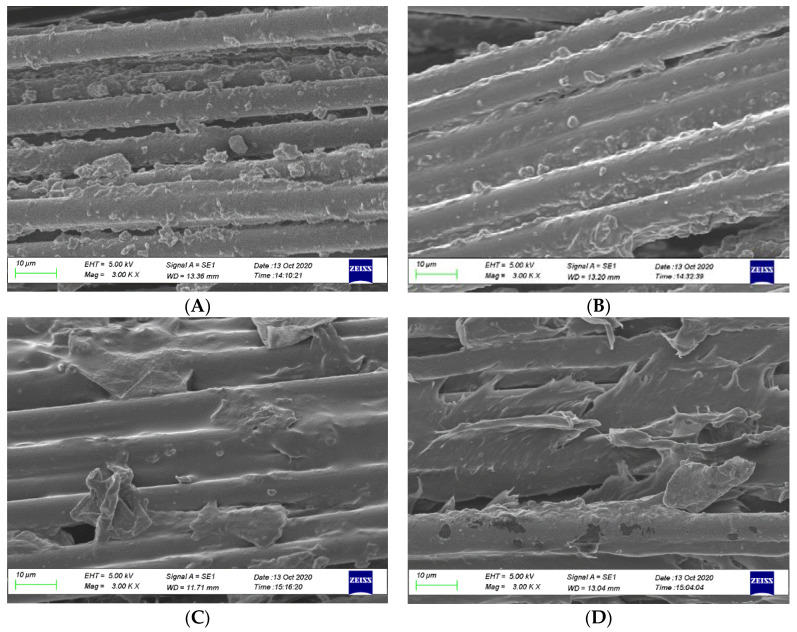
SEM micrographs of pull-out CF bundle at different processing parameters: (**A**) 210 °C/15 rpm/without pin/without treatment, (**B**) 210 °C/17 rpm/two pins/APTS, (**C**)190 °C/13 rpm/three pins/nitrogen and (**D**) 230 °C/13 rpm/three pins/nitrogen.

In addition, changes in impregnation quality due to the influences of process variables can be seen from SEM micrographs. The micrographs in Figure 19 were taken from the section of the specimens that have undergone a pull-out test where the matrix sleeve detached from the fibres. Figure 19A is a micrograph of the specimen produced at a melt temperature of 210 °C and pulling speed of 47 cm/min and without spreader pin and fibre treatment. The fibres are primarily bare on the fibre surface without resin residues attached; only a small amount of resin debris can be found on fibres exhibiting a poor interface on the smooth fibre surface. The figure shows that the impregnation quality is very low; therefore, the resulting IFSS is only 3.4 MPa. Figure 19B was taken from Trial 5 with the following process variables; melt temperature of 230 °C, pulling speed of 54 cm/min, two spreader pins and fibre treatment with VTMS. The impregnation quality increases, which is indicated by increased matrix adhesion to fibres; however, this combination only resulted in an IFSS of 5.17 MPa. The high pulling speed and the use of VTMS led to the low IFSS value. The fibre condition after the pull-out for the confirmatory test specimens is shown in Figure 19C,D. The figures show that better bonding conditions occur with a high number of spreader pins combined with the nitrogen treatment of fibres and low pulling speeds. The fibre is mostly coated by polypropylene resin together with a significant amount of debris on and between the fibres, providing evidence for better adhesion. Moreover, by applying a high melt temperature, plastic melt penetration into fibre gaps becomes easier, resulting in the maximum IFSS. Figure 19D shows that the increased penetration of the matrix layer provides shear resistance by improving fibre–matrix adhesion, efficiently transferring the load from the fibres to the matrix and avoiding the peeling off of CFs.

**Figure 20:**
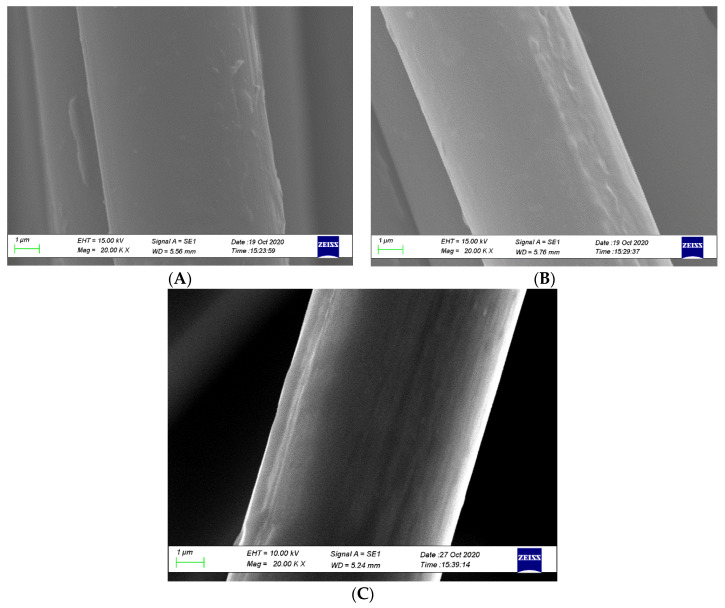
The surface roughness of treated carbon fibres: (**A**) VTMS treatment, (**B**) APTS treatment and (**C**) liquid nitrogen treatment.

As previously described in ANOVA, the fibre surface treatment (D) is the most significant parameter for the response. The objective of surface treatment is to increase the number of reactive functional groups or the surface roughness of the fibre to increase physical interactions with the matrix. In Figure 20, SEM micrographs show the surfaces of the treated fibres to compare their surface morphologies. As confirmed by other test results, liquid nitrogen provides better impregnation compared to VTMS and APTS. The micrographs (Figure 20A) show that VTMS-treated fibres have few surface defects and do not widely spread, indicating low surface roughness. Some microcavities and longitudinal shallow grooves are detected in the APTS fibre surface (Figure 20B), and these surface defects are localised. Consequently, the mechanical interlocking ability is better than VTMS treatment. Figure 20C shows that liquid nitrogen treatment increases the surface roughness of the CF, which can be observed by a remarkable increase in striations and skin fragments that widely spread on the fibre surface. The surface condition of CFs treated with liquid nitrogen looks coarser compared to that of CFs treated with VTMS and APTS; thus, liquid nitrogen is the best mechanical interlocking method for CFs due to the highest CF surface roughness attained.

## 4. Conclusions

In this study, Box–Behnken RSM was used to determine the effect of four processing variables of the extrusion–pultrusion process (melt temperature, pulling speed, number of spreader pins and fibre treatment) on the impregnation quality of CFRP filament composite. The following results were obtained.

- ANOVA showed that fibre treatment is the most influential parameter on IFSS, followed by pulling speed.
- The melt temperature in the quadratic order also shows a significant effect on IFSS. With the right combination of parameters, either high or low temperatures can lead to high IFSS.
- Treatment with liquid nitrogen is recommended for CFs because it increases the surface roughness, thereby providing a better matrix–fibre bonding effect.
- The optimisation step performed using two-parameter combination produced higher IFSS that the optimisation performed using the initial 27 test combination. IFSS of 24.2 MPa was obtained by using a melt temperature of 190 °C, pulling speed of 40 cm/min, three spreader pins and liquid nitrogen treatment, while an IFSS of 28.3 MPa was achieved using a melt temperature of 230 °C, pulling speed of 40 cm/min, three spreader pins and liquid nitrogen treatment.
- To achieve geometric stability, processing at a low temperature is essential to obtain optimum IFSS.

## Figures and Tables

**Table 1:** Material properties.

Material	Properties	Values
CF (T700SC 12K)	Filament diameter (µm)	7
	Density (g/cm^3^)	1.8
	Tensile strength (MPa)	4900
Cosmoplene AW564-PP	Density (g/cm^3^)	0.9
	Cylinder temperature (°C)	190–230
	Tensile strength at yield (MPa)	27.5
	Tensile strength at break (MPa)	23
	Melt Flow Index (g/10 min)	10
Vinyltrimethoxysilane (VTMS)	Density (g/cm^3^)	0.978
	Appearance	Colourless, transparent Liquid
γ-aminopropyl triethoxysilane (APTS)	Density (g/cm^3^)	0.9450–0.9550
	Appearance	Colourless, transparent Liquid
Liquid nitrogen	Boiling point (°C)	−196
	Density, Liquid @ BP, 1 atm (kg/m^3^)	808.5
	Specific Gravity, Liquid (water = 1) @ 20 °C, 1 atm	0.808

**Table 2:** Processing factors and levels.

Factors	Factor Codes	Actual Levels		
		Low (−1)	Middle (0)	High (+1)
Melt Temperature (°C)	A	190	210	230
Pulling speed (cm/min)	B	40	47	54
Number of spreader pins	C	1	2	3
Fibre treatment *	D	1	2	3

* 1: VTMS 1%; 2: APTS 1%; 3: Nitrogen.

**Table 3:** Three-level four-factor experimental design.

Run	Code				Actual			
	A	B	C	D	A	B	C	D
1	1	1	0	0	230	54	2	2
2	0	0	0	0	210	47	2	2
3	0	0	0	0	210	47	2	2
4	0	1	0	−1	210	54	2	1
5	−1	0	0	1	190	47	2	3
6	0	0	1	−1	210	47	3	1
7	0	−1	−1	0	210	40	1	2
8	1	0	1	0	230	47	3	2
9	−1	0	0	−1	190	47	2	1
10	0	0	0	0	210	47	2	2
11	1	0	0	−1	230	47	2	1
12	−1	1	0	0	190	54	2	2
13	1	0	−1	0	230	47	1	2
14	0	0	−1	1	210	47	1	3
15	0	1	0	1	210	54	2	3
16	0	1	1	0	210	54	3	2
17	1	−1	0	0	230	40	2	2
18	0	−1	0	−1	210	40	2	1
19	0	0	1	1	210	47	3	3
20	0	1	−1	0	210	54	1	2
21	−1	−1	0	0	190	40	2	2
22	0	−1	1	0	210	40	3	2
23	0	0	−1	−1	210	47	1	1
24	−1	0	−1	0	190	47	1	2
25	−1	0	1	0	190	47	3	2
26	0	−1	0	1	210	40	2	3
27	1	0	0	1	230	47	2	3

**Table 4:** Interfacial properties of the composite filament.

Trial Nr	Specimen 1	Specimen 2	Specimen 3	Average IFSS (MPa)	Std. Dev.
	IFSS (MPa)	IFSS (MPa)	IFSS (MPa)		
1	5.3	5.7	5.2	5.4	0.2
2	8.7	6.5	9.5	8.2	1.3
3	9.4	8.4	7.0	8.3	1.0
4	5.1	5.6	4.8	5.2	0.4
5	10.5	10.2	14.7	11.8	2.0
6	6.7	7.1	5.3	6.3	0.8
7	7.2	5.2	6.7	6.4	0.8
8	12.0	12.1	12.5	12.2	0.2
9	11.9	12.6	11.2	11.9	0.6
10	6.4	7.2	5.2	6.3	0.8
11	9.6	8.7	10.4	9.6	0.7
12	11.9	10.5	10.6	11.0	0.6
13	9.2	10.7	10.3	10.1	0.6
14	10.7	8.9	11.9	10.5	1.2
15	8.5	12.0	10.4	10.3	1.5
16	8.2	7.6	6.0	7.3	0.9
17	14.0	17.4	16.6	16.0	1.5
18	6.8	9.0	6.5	7.4	1.1
19	11.8	13.2	12.5	12.5	0.6
20	12.3	10.0	8.7	10.3	1.5
21	12.0	13.0	13.4	12.8	0.6
22	10.35	11.36	11.30	11.0	0.46
23	9.5	14.2	11.6	11.8	1.9
24	9.2	9.9	8.3	9.1	0.7
25	17.7	18.1	15.4	17.1	1.2
26	28.1	17.7	21.1	22.3	4.3
27	24.8	19.7	16.7	20.4	3.3

**Table 5:** ANOVA for interfacial shear strength (IFSS).

Source	Sum of Squares	df	Mean Square	F Value	*p* Value	Remarks
Model	362.50	14	25.89	2.98	0.0327	significant
A-Melt Temperature	0.0005	1	0.0005	0.0001	0.9944	
B-Pulling speed	58.32	1	58.32	6.71	0.0237	significant
C-Number of pins	5.69	1	5.69	0.6543	0.4343	
D-Fibre treatment	105.89	1	105.89	12.18	0.0045	significant
AB	19.51	1	19.51	2.24	0.1599	
AC	8.54	1	8.54	0.9818	0.3413	
AD	30.00	1	30.00	3.45	0.0879	
BC	14.78	1	14.78	1.70	0.2167	
BD	23.78	1	23.78	2.74	0.1241	
CD	13.99	1	13.99	1.61	0.2287	
A²	62.99	1	62.99	7.25	0.0196	significant
B²	2.47	1	2.47	0.2838	0.6040	
C²	1.85	1	1.85	0.2133	0.6524	
D²	33.67	1	33.67	3.87	0.0726	
Residual	104.33	12	8.69			
Lack of fit	101.78	10	10.18	7.99	0.1163	not significant
Pure error	2.55	2	1.27			
Cor total	466.82	26				

**Table 6:** The statistical measurement from ANOVA for IFSS.

Source	Respond
R-Squared (R^2^)	0.7766
Adj R-Squared	0.5159
Pred R-Squared	−0.2676
Adequate Precision	6.2922

**Table 7:** Results of the confirmation test.

Variables				Response
A. Melt temperature (°C)	B. Pulling speed (cm/min)	C. Number of pins	D. Fibre treatment	Avg IFSS (MPa)
190	40	3	Liquid nitrogen	24.2
230	40	3	Liquid nitrogen	28.3
